# New Inventions

**Published:** 1912-08

**Authors:** 


### NEW INVENTIONS

F. R. Anderson, Providence, R. I. 1,030,861. Foot and Ankle Brace.

Georg Ornstein, Berlin, Ger. 1,030,916. Process of Producing Acetylene Tetrachlorid.

G. A. Peate and Thos. Beattie, Montreal, Que., Can. 1,030,964, 1,030,965. Device for Preventing and Curing Stammering and Stuttering.

A. A. Finta, Chicago, Ill. Assignor to The Child's Welfare Garment Co. 1,030,989. Infant's Garment.

W. G. Euston, St. Louis, Mo. 1,031,109. Resuscitating Apparatus.

Elise Conrad, Milwaukee, Wisc. 1,031,247. Antislipping Attachment for Crutches.

D. L. Aber, Pittsburgh, Pa. 1,031,314. Surgical Instrument.

Levey McCoy, Memphis, Tenn. 1,031,565. Embalming Apparatus.

Fred'k Soddy, Glasgow, Scotland. 1,031,593. Process of Making Crystals containing Mesothorium.

L. L. Zarbaugh, Toledo, Ohio. 1,031,609. Syringe-Nozzle Attachment.

Frank Kuhn, Detroit, Mich. Assignor to Amer. Electrical Heater Co., Detroit, Mich. 1,025,144. Electric Foot-Warmer.

E. M. Grindle, Holton, Kas. 1,025,265. Cheek-Distender.

Jas. Birrell and Wm. Birrell, Seattle, Wash. Assignors to Birrell Vacuum Vibrator Co., Seattle, Wash. 1,025,504. Combined Vibrator and Vacuum Apparatus.

C. A. Howe, Chicago, Ills. 1,025,571. Foot-Support.

T. J. Donovan, Syracuse, N. Y. 1,025,612. Bathing-Machine.

R. M. Machlett, New York, N. Y. 1,025,635. Gas-Regulator for Gas-Tubes.

D. M. Don Jian, Washington, D. C. 1,025,903. Device for Removing and Curing Corns.

H. W. Sanford, Washington, D. C. 1,025,207. Surgical Kit.

J. F. Craven, Pittsburgh, Pa. Assignor to Craven Engineer-
for containing and discharging semisolid and pasty substances.
for containing and discharging semisolid and pastry substances

Otto Schmidt, Mannheim, Ger. Assignor to Badisch Anlin & Soda Fabrik, Ludwigshafer-on-the-Rhine, Ger. 1,025,652. Chlor-Aralkyl-Sulfonic Acids and process of making such compounds.

Heinrich Hoerlein, Vohuxnkel, near Elberfeld, Ger. Assignor to Farbenfabriken vorm. Friedr, Bayer & Co. 1,025,872 Phenylethylbarbituric Acid.

Nathan Sulzberger, New York, N. Y. 1,025,889. Chloral Devivatives containing the Radical of a Fatty Acid.

Otto Liebknecht Frankfort-on-the-Main, Ger. Assignor to Roessler & Hasslacher Chemical Co., New York, N. Y. 1,025,948. Stable Hydrogen-Peroxid Solution.

Chas. DeBock, Lele, Belgium. 1,023,803. Sucking Bottle.

Arthur Dubay, Gardiner, Me. 1,023,822. Teething Finger.

Henry J. Smith, Philadelphia, Pa. 1,024,152 and 1,024,153. Method and Means for Producing Dental Plates.

Moritz Saenger, Madgeburg, Germany, 1,024,422. Medical Spraying Syringe.

G. J. Kelley, Attleboro, Mass. 1,029,689. Vaginal Syringe.

F. R. Kunkel, Edgewood Park, Pa. Assignor to Westinghouse Elec. & Mfg. Co. 1,029,696. Massage Apparatus.

J. E. MacWilliam, Hubbardston, Mass. Assignor to F. A. MacWilliam, Hubbardston, Mass. 1,029,813. Nasal Douche.

J. W. Bates, Hastings, New Zealand. 1,029,841. Evaporating and Fumigating Apparatus.

E. W. Caldwell, New York, N. Y. 1,029,250. X-Ray Tube.

H. C. Folger, Somerville, Mass. Assignor to Nu-Vo Mfg. Co. 1,029,861. Electric Hair and Scalp Treating Instrument.

Mose Wilbuschewitsch, Nischniovgerod, Kanairno, Russia. 1,029,901. Process of Manufacturing Catalysts.

K. L. Storm, Philadelphia, Pa. 1,029,955. Abdominal Supporter.

Alek Bauer, Chicago, Ill. Assignor to Bauer & Black, Chicago, Ill. 1,030,224. Convertible Suspensory and Supporter.

C. P. Beckwith, Detroit, Mich. Assignor to Parke, Davis & Co., Detroit, Mich. 1,030,378. Appartus for Discharging Liquids in measured quantities.

L. A. Saunders, Los Angeles, Cal. 1,030,543. Syringe with Electric Attachment.

J. M. White, Danville, Va. 1,130,696. Bottle-Indicator.

Geo. E. Graham, Candor, N. Y. 1,024,506. Combination Reflecting Means for Enabling one to see the back of the head and the back of the neck.

F. C. Searle, Wilmington, Del. 1,024,527. Burial Vault.

Graham Clark, Cleveland, Ohio. 1,024,556. Attachment for Anesthetizing Machines.

Claes W. Boman, New York, N. Y. 1,024,824. Clinical Thermometer Casing.

J. M. Baber, Henrietta, N. C. 1,024,966. Operating Table.

L. L. Miner, Bisbee, Ariz. 1,025,008. Brace for Fractured Bones.

Elsie R. Norwood, San Francisco, Cal. 1,025,012. Water Bag Retiner.

B. S. Lacy, Dubuque, Iowa. 1,031,720. Nursing Bottle Nipple.

T. M. Merck, Gainsville, Ga. 1,031,724. Truss.

W. H. Eblen, Windrock, Tenn. 1,031,841. Obsteric Appliance.

Mary J. F. Grundy, Chicago, Ill. 1,031,861. Antichafing and Menstrual Garment.

J. B. Young, Cumberland, Ind. 1,031,901. Thermometer-Case.

G. E. Marks, New York, N. Y. Assignor to The Firm of A. A. Marks, New York, N. Y. 1,032,074. Artificial Leg.

W. S. Hay, Providence, R. I. Assignor to Davol Rubber Co., Providence, R. I. 1,032,333. Automizer.

